# Immunization with a novel mRNA vaccine, TGGT1_216200 mRNA-LNP, prolongs survival time in BALB/c mice against acute toxoplasmosis

**DOI:** 10.3389/fimmu.2023.1161507

**Published:** 2023-04-14

**Authors:** Yizhuo Zhang, Dan Li, Yu Shen, Shiyu Li, Shaohong Lu, Bin Zheng

**Affiliations:** ^1^Institute of Parasitic Diseases, School of Basic Medicine and Forensics, Hangzhou Medical College, Hangzhou, China; ^2^Engineering Research Center of Novel Vaccine of Zhejiang Province, Hangzhou Medical College, Hangzhou, China; ^3^Key Laboratory of Bio-tech Vaccine of Zhejiang Province, Hangzhou Medical College, Hangzhou, China

**Keywords:** Toxoplasma gondii, vaccine, mRNA, lipid nanoparticle, immune response

## Abstract

*Toxoplasma gondii*, a specialized intracellular parasite, causes a widespread zoonotic disease and is a severe threat to social and economic development. There is a lack of effective drugs and vaccines against *T. gondii* infection. Recently, mRNA vaccines have been rapidly developed, and their packaging materials and technologies are well established. In this study, TGGT1_216200 (TG_200), a novel molecule from *T. gondii*, was identified using bioinformatic screening analysis. TG_200 was purified and encapsulated with a lipid nanoparticle (LNP) to produce the TG_200 mRNA-LNP vaccine. The immune protection provided by the new vaccine and its mechanisms after immunizing BABL/C mice *via* intramuscular injection were investigated. There was a strong immune response when mice were vaccinated with TG_200 mRNA-LNP. Elevated levels of anti-*T. gondii*-specific immunoglobulin G (IgG), and a higher IgG2a-to-IgG1 ratio was observed. The levels of interleukin-12 (IL-12), interferon-γ (IFN-γ), IL-4, and IL-10 were also elevated. The result showed that the vaccine induced a mixture of Th1 and Th2 cells, and Th1-dominated humoral immune response. Significantly increased antigen-specific splenocyte proliferation was induced by TG_200 mRNA-LNP immunization. The vaccine could also induce *T. gondii*-specific cytotoxic T lymphocytes (CTLs). The expression levels of interferon regulatory factor 8 (IRF8), T-Box 21 (T-bet), and nuclear factor kappa B (NF-κB) were significantly elevated after TG_200 mRNA-LNP immunization. The levels of CD83, CD86, MHC-I, MHC-II, CD8, and CD4 molecules were also higher. The results indicated that TG_200 mRNA-LNP produced specific cellular and humoral immune responses. Most importantly, TG_200 mRNA-LNP immunized mice survived significantly longer (19.27 ± 3.438 days) than the control mice, which died within eight days after *T. gondii* challenge (*P<* 0.001). The protective effect of adoptive transfer was also assessed, and mice receiving serum and splenocytes from mice immunized with TG_200 mRNA-LNP showed improved survival rates of 9.70 ± 1.64 days and, 13.40 ± 2.32 days, respectively (*P<* 0.001). The results suggested that TG_200 mRNA-LNP is a safe and promising vaccine against *T. gondii* infection.

## 1 Introduction

*Toxoplasma gondii*, as an obligate intracellular apicomplexan protozoan, is capable of infecting almost all warm-blooded animals, and causes zoonotic infections in humans. It is generally assumed that *T. gondii* infects approximately 25% to 30% of the world’s human population. Latin America and tropical African countries have higher prevalences of *T. gondii* infection (1, 2). Infection in humans usually occurs *via* ingesting tissue cysts in infected meat or ingesting water or food contaminated with sporulated oocysts produced in the environment. Besides, some infections are iatrogenic and congenital and are transmitted vertically.

Toxoplasmosis is a common opportunistic infection, and primary infection is usually subclinical; however, cervical lymphadenopathy or eye disease might be present in some patients. It can cause ocular toxoplasmosis and even schizophrenia and bipolar disorders by accumulating in the central nervous system (3–5). Immunocompromised individuals (patients with AIDS or recipients of organ transplants) or pregnant women infected with *T. gondii* would develop significant clinical symptoms (6). Infection of pregnant women can be detrimental to the fetus and even lead to stillbirth. The gold standard for treating toxoplasmosis is the combination of pyrimethamine and sulfadiazine, which can inhibit the synthesis of parasite DNA by inhibiting dihydrofolate reductase (DHFR) (7). However, this treatment can cause folate deficiency states, which can be responsible for significant side effects on the blood system and embryonic diseases, in addition to the risk of causing diseases such as agranulocytosis and Stevens-Johnson syndrome (8). Vaccination for disease prevention is the best way to control diseases, for example, human papillomavirus vaccines and COVID-19 vaccines; therefore, the development of toxoplasmosis vaccine candidates is imperative (9, 10).

Among several candidate vaccines under investigation, including live attenuated, DNA, mRNA, epitope, carbohydrate-based, and subunit vaccines, only one vaccine, classified as a live attenuated vaccine, has been commercially licensed and used in the sheep industry: Toxovax® (MSD, Wellington, New Zealand). It was obtained from the non-cyst-forming S48 strain. Until now, there have been no other vaccines with significant protective efficiency for commercial licensing (11–13). Furthermore, these vaccines still have some shortcomings. For example, attenuated vaccines can be counterproductive due to their unknown genetic background, causing diseases they are supposed to prevent because of the possibility of reversion to the toxic wild-type. Moreover, the integration of potential genomic plasmids of DNA vaccines can activate relevant proteins and produce antibodies against the vaccine itself. Epitope vaccines lack the secondary and tertiary structures of the native proteins and are small molecules with poorly immunogenicity. In carbohydrate-based vaccines, the structure of the carbohydrate tends to be similar to that of the host, which might lead to autoimmunity. Carrier or adjuvant delivery is usually required for subunit vaccines, but this provides less immune protection (12, 14). mRNA vaccines theoretically avoid the risk of genetic recombination as they will be confined to the cytoplasm. Moreover, they are relatively safe, with little risk of misfolding recombinant proteins. In addition, independent cell expansion shortens production time and is more conducive to preventing epidemic diseases (15). The COVID-19 mRNA vaccine was developed and has been used in clinical settings in just two years, showing the possibility of developing a *T. gondii* mRNA vaccine (16).

In this study, BALB/c mice were used as the animal models for immunization to study the immunogenicity and protective efficiency of a novel vaccine, comprising TGGT1_216200 (TG_200) mRNA, a novel molecule from *T. gondii*, encapsulated in a lipid nanoparticle (TG_200 mRNA-LNP vaccine). We evaluated antibody levels, cytokine levels, and immune-related transcription factor levels. The lymphocyte proliferation and cytotoxic T lymphocyte (CTL) activity were also assessed. Survival time was analyzed after *T. gondii* RH strain challenge, including vaccine-immunized mice and mice receiving adoptive transfer. Herein, the potential mechanisms and prospects of the vaccine were also discussed.

## 2 Materials and methods

### 2.1 Epitope prediction

Bioinformatic prediction was carried out using DNASTAR software (Madison, WI, USA) and the Immune Epitope Database (IEDB, http://tools.immuneepitope.org/mhcii/). Protean software in the DNASTAR package predicted the biochemical indicators (surface probability, hydrophilicity, flexibility, and antigenicity), and the half-maximum inhibitory concentration (IC50) value of a peptide that bound to the major histocompatibility complex (MHC)-II molecule TG_200 was analyzed in the IEDB. Applying the IEDB methodology, the prediction results were ranked by percentiles based on the suggested methodology.

### 2.2 Animals

BALB/c mice (6–8 weeks old) and New Zealand white rabbits (7–9 weeks old) were purchased from the Zhejiang Experimental Animal Center (Zhejiang, China). All animals were kept in standard pathogen-free conditions with stable temperatures and were used for the vaccination study. The procedures in this study involving the mice and rabbits were performed in accordance with Chinese legislation on the use and care of laboratory animals (GB/T35823-2018). The animal experiments were approved by Hangzhou Medical College Institutional Animal Care and Use Committee (Approval No: 2021-152). Regarding euthanasia, mice were euthanized by intraperitoneal injection of sodium pentobarbital (150 mg/kg).

### 2.3 Parasites and cells

We used the human foreskin fibroblast (HFF) cells to maintain and proliferate the *T. gondii* RH strain (type I) tachyzoites. The obtained tachyzoites were used to prepared the soluble tachyzoite antigens (STAg). Furthermore, the tachyzoites were used for total RNA extraction, and in the mice challenge experiments. HFF cells, cultured muscle cells (C2C12), 293T cells from the human embryonic kidney (293T cells), and a strain of *T. gondii* RH were stored at −80°C in our laboratory. 293T and C2C12 cells were used for the transfection experiments, which used the lipo2000 reagent (Invitrogen, Carlsbad, CA, USA). The HFF, 293T, and C2C12 cells purchased from American Type Culture Collection (ATCC; Manassas, VA, USA) and maintained in Dulbecco’s modified Eagle’s medium (DMEM) containing 10% fetal bovine serum (FBS; Gibco, New Zealand) in an incubator with 5% CO_2_ at 37°C.

### 2.4 Preparation of rabbit anti-TG_200 polyclonal antibodies

Using the Trizol reagent (Invitrogen), we extracted the total RNA of *T. gondii* from the tachyzoites of the RH strain, according to the manufacturer’s protocol. The GoScript™ Reverse Transcription System (Promega, Madison, WI, USA) was used to synthesize cDNA from the total RNA. The whole TG_200 open reading frame (ORF) was amplified from the *T. gondii* RH tachyzoite cDNA using PCR with the following primers: forward: 5’-CGGGATCCATGGCGTTAACCATGTTGGCA-3’; reverse: 5’-CCAAGCTTTCAGGCGGCCTACTGGCCGTGCAC-3’, which were designed using the DNASTAR software and incorporated BamHI and HindIII restriction sites. The recombinant plasmid pET28a-TG_200 was constructed by ligating the PCR product into vector pET-28a *via* the BamHI/HindIII sites. *Escherichia coli* BL21(DE3) was chosen to express recombinant TG_200 (rTG_200), which was purified using Ni^2+^-NTA agarose columns (Qiagen, Hilden, Germany).

Two rabbits were immunized with 200 μg of purified rTG_200 protein intravenously to obtain polyclonal antiserum. Three injections in each rabbit were administered at intervals of two weeks. We collected blood from the rabbit ear vein the day before each injection.

### 2.5 Design of the TG_200 mRNA construct and *in vitro* transcription (IVT)

**Figure 1:**
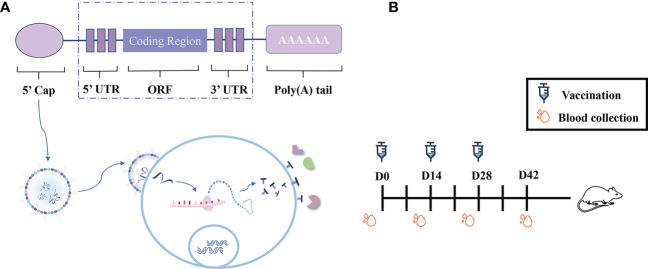
Experimental design and immunization schematic diagram. **(A)** Schematic diagram of the construction of TG_200 mRNA-LNP vaccine candidates and a schematic diagram showing the proposed mechanism for mRNA vaccine candidate translation in the cytoplasm. **(B)** Scheme of the TG_200 mRNA-LNP vaccination schedule.

The mRNA construct comprised five structures to make it more complete and stable, including a 5′ cap structure, a 5′ untranslated region (UTR), the open reading frame (ORF) of TG_200, a 3′ UTR, and a Poly-A tail (**Figures 1A**, **S1**). The 300 bp 5′ UTR (the sequence was chosen by searching in ToxoDB http://toxodb.org/toxo/) was placed before the TG_200 coding sequence, and a 300 bp sequence was placed after the TG_200 coding sequence as the 3′ UTR. The T7 promoter was chosen for *in vitro* transcription of the mRNA, and the modified mRNA was synthesized from a linearized DNA template *in vitro* using a T7-FlashScribe Transcription Kit (CellScript, Madison, WI, USA). The 5′ cap and poly-A tail structure were added enzymatically, contributing to the recognition and binding of the mRNA by ribosomes and the stabilization of the mRNA structure during protein expression. To perform IVT, standard unmodified nucleotides were used as described by the manufacturer. We generated mRNA for the encapsulation in LNPs after replacing uridine with pseudouridine using the Incognito mRNA synthesis kit (CellScript). The mRNA was stored frozen at −80°C until use.

### 2.6 Generation of mRNA/LNP and encapsulation efficiency

The mRNA-LNP formulation was prepared using a GenVoy ionizable lipid mixture (GenVoy-ILM) (Precision NanoSystems (PNI), Santa Rosa, CA, USA) on the benchtop. GenVoy-ILM is the simplest way to make LNPs. GenVoy-ILM is a mixture of molar ratio 50 (PNI Ionizable Lipid):10 Distearoylphosphatidylcholine (DSPC):37.5 (cholesterol):2.5 (PNI Stabilizer). The program was set with a total flow rate of 12 mL/min and a flow ratio of 3:1 to pass the mRNA and GenVoy ILM in PNI buffer through a laminar tube that encapsulates lipids to generate mRNA-LNPs. As described by the manufacturer, the mRNA encapsulation efficiency was calculated using a quantit™ RiboGreen™ RNA Reagent and Kit (Thermo Fisher Scientific, Waltham, MA, USA). The fluorescence intensity was converted to concentration using a standard curve drawn using standard solutions. Finally, the encapsulation efficiency (EE) was calculated according to the following formula (in which “W” represents weight): EE% = W(*total* RNA)-W(*free* RNA)/W(*total* RNA).

### 2.7 mRNA/LNP delivery *in vitro*

According to the manufacturer’s instructions, the mRNA synthesized *in vitro* was transfected into 293T cells using the lipo2000 reagent, ensuring the correct translation of mRNA in the cell. The skeletal muscle myoblast C2C12 cells were transfected to ensure that the synthesized mRNA could be expressed by intramuscular injection. The collected cell lysate and supernatant were analyzed using western blotting with rabbit anti-TG_200 polyclonal antibodies. *In vitro* synthesized mRNA (pseudouridine in place of uridine) was purified and encapsulated in LNPs for administration to mice.

### 2.8 Immunization and *T. gondii* challenge

Female BALB/c mice were randomly divided into three groups of 10 mice per group. Ten mice were immunized with the TG_200 mRNA-LNP vaccine three times intramuscularly in a 100 μL volume (1 mg/mL) as an experimental group (**Figure 1B**). Ten mice were injected with LNP as negative control and the rest, without treatment, formed the BLANK control group. Before each immunization and three weeks after the final injection, we collected serum samples from all mice. Two weeks after the last injection, each group was challenged with 10^2^ tachyzoites of the RH strain. The mice challenged by RH *T. gondii* were observed and their survival was recorded.

### 2.9 Indirect enzyme-linked immunosorbent assay (ELISA)

Before blood collection, the tail of the mice was placed in water at about 45°C to fill up the tail vessels. The tail was wiped with 75% alcohol, a transverse incision was made using a razor blade to cut through the caudal vein, and about 0.1 mL of blood was taken. Finally the wound was disinfected and pressure was applied to stop the bleeding. Total IgG and subtype antibodies (IgG1, IgG2a) in mice serum after TG_200 mRNA-LNP immunization were determined indirectly using ELISA with 10 ​μg/mL rTG_200 protein-coated flat-bottom 96-well plates in bicarbonate buffer (pH 9.6) at 4 ​°C overnight. The coated plates were blocked with 5% skim milk in phosphate-buffered saline with 0.05% Tween 20 (PBST) for 1 h at 37°C, washed with PBST three times, then incubated with 100 μL of serum samples (1:100 in PBST) for 2 h at 37°C after adding, followed by incubation with 100 μL of horseradish peroxidase (HRP)-conjugated goat-mouse IgG, IgG1 or IgG2a (Abcam, Waltham, MA, USA) for 1 h at 37°C. The plates were then washed three times and incubated with 100 μL of tetramethylbenzidine (TMB) for 10 min. Then, 50 μL of 2 M H_2_SO_4_ was added to stop the reaction. An automatic ELISA reader (BioTek, Winooski, VT, USA) was used to detect the absorbance values at 450 nm. Each sample contained three replicates.

### 2.10 Lymphocyte proliferation assay

Splenic lymphocytes were removed from five mice in each group after euthanasia at two weeks after final immunization. The spleen was pushed through a nylon sieve to harvest the spleen cells. To remove the red blood cells, red blood cell lysis buffer (Sigma, St. Louis, MO, USA) was used. Moreover, DMEM, which was supplemented with 10% fetal bovine serum, was used to resuspend the purified spleen cells. The purified spleen cells were cultured in 96-well plates (2 × 10^5^ cells/well) in triplicate in the presence of 10 μg/mL rTG_200 protein. The negative control comprised cells incubated in complete medium, and the positive control comprised cells incubated in 5 μg/mL concanavalin A (ConA). The plates were incubated for four days at 37°C under 5% CO_2_. The proliferative activity of spleen lymphocytes was detected using the Cell Counting Kit-8 (CCK-8) reagent (Dojindo, Kumamoto, Japan) following the manufacturer’s protocol. The absorbance was read and recorded at 570 nm. Then, the stimulation index (SI) was calculated using the following formula:


$$\text{SI}=\left({\text{OD}}_{570}\text{rTG}\_200/{\text{OD}}_{570}\text{Control}\right):\left({\text{OD}}_{570}\text{ConA}/{\text{OD}}_{570}\text{Control}\right).$$

### 2.11 Detection of cytokines

Following a previously described protocol, five mice spleens were taken from each group to detect cytokine levels at two weeks after the last injection (17). Spleen cells in 96-well plates were cultured at 37°C, 5% CO_2_ and cell-free supernatants were collected. Interleukin (IL)-4 levels at 24 h, IL-10 levels at 72 h, and interferon-gamma (IFN-γ) and IL-12 levels at 96 h were detected using commercially available kits (eBioscience, San Diego, CA, USA) according to the manufacturer’s instructions. All samples were assayed three times.

### 2.12 Flow cytometry

Splenocytes from five mice per group were isolated two weeks after the last immunization. The isolated lymphocytes were then cultured in DMEM with10% fetal bovine serum and 1% penicillin-streptomycin overnight. The nonadherent cells were removed and the remaining cells were collected in PBS after three washes. 10^6^ cells were conditioned in 100 μL of PBS and stained with anti-mouse CD11c-fluorescein isothiocyanate (FITC), CD83-Phycoerythrin (PE), and CD86-PE (eBioscience) at 4°C in dark conditions for 40 min. Flow cytometry (Beckman Coulter Inc., Brea, CA, USA) was used to sort and count cells after washing and collection to investigate the surface markers of dendritic cells (DCs). Consistent with the above method, the molecular changes of MHCs in DCs were analyzed using anti-mouse CD11c-FITC, MHC-I-PE, and MHC-II-PE (eBioscience). After adjusting the cell numbers, two cell tubes were collected and respectively stained with anti-mouse CD3e-FITC, CD4-PE (eBioscience) and anti-mice CD3e-PE, CD8-PE to detect the subsets of CD4^+^ T and CD8^+^ T lymphocytes. The ratio of CD4^+^ and CD8^+^ T lymphocyte subsets was also analyzed. All samples were assayed in triplicate by performing the staining strategy and flow cytometry analysis. All labeled cells were analyzed on a FACSAria III flow cytometer (BD Biosciences, Franklin Lakes, NJ, USA), and the data were analyzed using CellQuest software (BD Biosciences).

### 2.13 Quantitative real-time reverse transcription PCR (qRT-PCR)

The rverse transcription step of the qRT-PCR protocol was consistent with the preparation of polyclonal antibodies as described in section 2.4. Using CFX96 connect apparatus (Bio-Rad, Hercules, CA, USA), the qPCR step of the qRT-PCR protocol was caried out using the prepared cDNA as the template. The reactions were conducted in triplicate using intercalating dye SYBR Green-based GoTaq^®^ qPCR Master Mix (Promega), following the manufacturer’s instructions. The experimental results were normalized by selecting H3 (encoding Histone H3) as an internal reference gene. The primers used are listed in **Table 1**.

**Table 1:** qRT-PCR primers used to amplify the *p65*, *Tbet*, *Irf8*, and *Actb* genes designed by DNASTAR software.

Primer name	Sequence
NF-κB p65-F	5′-GAACCAGGGTGTGTCCATGT -3′
NF-κB p65-R	5′-TCCGCAATGGAGGAGAAGTC-3′
T -bet-F	5′-GCCAGGGAACCGCTTATATG-3′
T -bet-R	5′-TGGAGAGACTGCAGGACGAT -3′
IRF8-F	5′-GCTGATCAAGGAACCTTGTG-3′
IRF8-R	5′- CAGGCCTGCACTGGGCTG−3′
β-Actin-F	5′-GCTTCTAGGCGGACTGTTAC-3′
β-Actin-R	5′-CCATGCCAATGTTGTCTCTT -3′

### 2.14 Western blotting analysis

The expression of the TG_200 protein in 293T cells and C2C12 cells were analyzed by western blotting (WB). The level of β-actin in these cell lysates served as an internal control. Detection of TG_200 was performed using rabbit anti-TG_200 polyclonal antibodies as the primary antibodies. Moreover, WB was used to validate the levels of downstream molecules T-Box 21 (T-bet), interferon regulatory factor 8 (IRF8), and the nuclear factor kappa B (NF-κB) p65 subunit. Antibodies against T-bet, p65, and IRF8 were purchased from Cell Signaling Technology, Inc. (Danvers, MA, USA). The nuclear and cytoplasmic isolation kit (Beyotime Institute, Haimen, China) was used to isolate transcription factors (NF-κB p65, T-bet, and IRF8) in the nucleus, which were assessed using WB. The antibody was diluted with 0.05% skim milk in a ratio of 1:1000. The detailed assay steps refer to a previous publication (17).

### 2.15 Cytotoxic T lymphocyte activity assays

A CytoTox 96^®^ Non-Radioactive Cytotoxicity Assay Kit (Promega) measured the CTL activity. The spleen cells were cultured with 100 U/mL recombinant murine IL-12 for five days as effector cells. The target cells were Sp2/0 mice cells that were transfected with TG_200 mRNA-LNP. Effector and target cells were mixed at ratios of 10:1, 20:1, 40:1, and 80:1. The percentage of specific cell lysis was calculated at 6 h after incubation as follows: Cytotoxicity % = (Experimental-Effector Spontaneous-Target Spontaneous)/(Target Maximum-Target Spontaneous) × 100.

### 2.16 Adoptive transfer study

Sera from three groups of mice (TG_200 mRNA-LNP, LNP, and BLANK) were collected and transferred to naive mice by tail vein injection (100 µL per mice) to assess the protective effect of the immune sera. Thirty mice were randomly divided equally into three groups, TG_200 mRNA-LNP (TG_200 immunized serum injection), LNP (LNP immunized serum injection), BLANK (BLANK serum injection), respectively. After five daily transfers, each group was challenged with 10^2^ tachyzoites of the RH strain. Survival time was recorded after the mice were challenged with *T. gondii* and their survival was monitored daily.

Splenocytes from the three groups of mice were collected and transferred to naive mice by tail vein injection (5 × 10^7^ cells mice) to assess the protective of the immune splenocytes. Thirty mice were randomly divided equally into three groups, TG_200 mRNA-LNP (TG_200 immunized splenocytes injection), LNP (LNP immunized splenocytes injection), BLANK (BLANK splenocytes injection), respectively. Each group was challenged with 10^2^ tachyzoites of the RH strain at 24 h after transfer. Survival time was recorded after the mice were challenged with *T. gondii* and their survival was monitored daily.

### 2.17 Statistical analysis

An independent sample T-test was performed for the levels of antibodies, cytokines, lymphocyte proliferation, flow cytometry analysis; and a Log-rank test was for survival analysis in GraphPad 8.0 software (GraphPad Inc., San Diego, CA, USA). Differences were considered statistically significant when *P*<0.05.

## 3 Results

### 3.1 Bioinformatic analysis identifies B- and T-cell epitopes in TG*_*200

**Figure 2:**
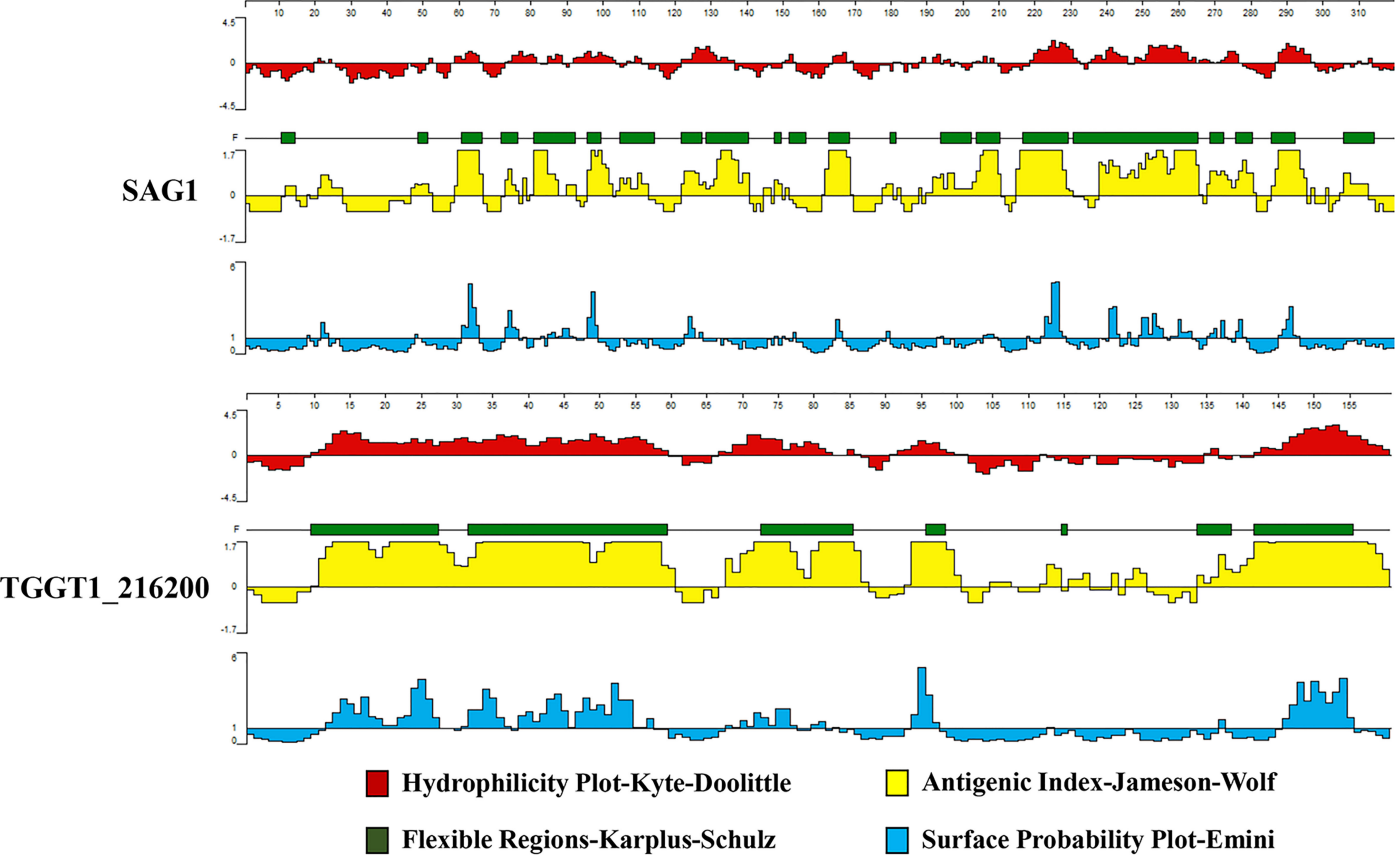
The plot of the DNASTAR-predicted hydrophilicity, flexible regions, antigenic index, and surface probability of the linear-B cell epitopes of TG_200 compared with those of SAG1.

Numerous antigens have been identified as potential vaccines against *T. gondii*, such as surface antigen proteins (TgSAGs), rhoptry protein (TgROPs), microneme proteins (TgMICs), and others (18). Despite choosing diverse immunization routes, animal models, doses, and vaccine production processes, none of the vaccines developed based on these candidates have demonstrated complete protection against *T. gondii*. Secreted proteins play a crucial role in mediating the interaction between host and pathogen (19); therefore we focused on analyzing *T. gondii* secreted proteins at the genome-wide level to find possible vaccine candidates. Using the ToxoDB, we first screened secreted proteins containing both transmembrane domain and signal peptide at the whole-genome level. Out of over 8,000 genes in the *T. gondii* genome, only 233 genes had both transmembrane domains and signal peptides. A vaccine candidate with a high potential for success is often identified by a bioinformatic analysis that predicts a high T/B cell epitope score (20, 21). Based on published data, *T. gondii* surface antigen 1 (SAG1) has strong immunogenicity and immunoprotective effects, and is considered a promising candidate antigen and a major target for inducing host immune responses (22). Therefore, SAG1 was chosen as the reference protein for this experiment. We used DNASTAR (PROTEAN program) and IEDB database to perform T/B cell epitope analysis on the 233 genes encoding proteins that were screened, and compared them with SAG1. We obtained several candidate proteins, among which TGGT1_216200 proteins showed the best T/B cell epitope prediction scores. TG_200 and SAG1 scores for surface probability, antigenic index, hydrophilicity, and flexible regions were compared. Flexible amino acid residues are malleable sites that readily form antigenic epitopes. Hydrophilic amino acids are enriched on the surface of hydrophilic regions, and these sites have evolved to become the primary amino acid insertion sites for proteins, which also provide the basis for protein secretion into the cytoplasm and extracellular compartments. Surface probability indicates the probability that an antigenic site is located on an exposed region of the protein surface. The antigenic index can reflect the antigenicity scale by analyzing amino acids at contiguous sites of well-studied proteins. According to **Figure 2**, in comparison with SAG1, each TG_200 indicator showed a high score. The regions with hydrophilicity of TG_200 are amino acids 10–60, 67–86, 92–102, 135–137, and 142–160. The surface probability regions of TG_200 are amino acids 13–27, 32–36, 39–54, 57, 68–77, 80–82, 94–97, 137, 147–156. The antigen index regions of TG_200 are amino acids 11–60, 68–78, 80–87, 93–100, 113–114, 116–118, 122, 124–126, 135–160. The flexibility regions of TG_200 are amino acids 10–27, 32–59, 73–85, 96–98, 115, 134–138, 142–155. The TG_200 protein has good surface probability, hydrophilicity, a high antigenic index, and more variable regions, indicating that vaccines based on TG_200 protein might be promising.

In addition, both TG_200 and SAG1 T-cell epitopes were analyzed using the IEDB online service. The MHC II molecules were correlated with the half-maximum inhibitory concentration (IC50) values of the TG_200 peptides, and generally, the smaller the value, the stronger the epitope and the binding ability of MHC-II. **Table 2** shows that there are lower percentile classes of TG_200 peptides than those of SAG1. The IC50 values of H2-IAd, H2-IEd, and HLA-DRB1*01:01 from TG_200 were lower than those of SAG1. This result indicated that the TG_200 protein might have strong binding to MHC-II.

**Table 2:** IC50 values for TG_200 and SAG1 binding to MHC class II molecules, obtained using IEDB^a^.

MHC II Allele^b^	Start-Stop^c^		Percentile Rank^d^	
	SAG1	TG_200	SAG1	TG_200
**H2-IAb**	26-40	114-128	0.95	1.20
**H2-IAd**	21-35	5-19	2.85	1.12
**H2-IEd**	14-28	207-221	3.35	0.44
HLA-DRB 1*01:01	12-26	180-194	1.80	0.50

a The Immune Epitope Database (http://tools.immuneepitope.org/mhcii).

b H2-IAb, H2-IAd, and H-2-IEd alleles are mouse MHC class II molecules; the HLA-DRB 1*01:01 allele is a human MHC class II molecule.

c 15 amino acids were chosen for analysis.

d Low percentile indicates high level binding according to the software instructions.

The bioinformatic analysis showed that the TG_200 peptide has a higher linear B-cell epitope score and lower IC50 value percentile compared with those of SAG1, suggesting that TG_200 might be more promising as a candidate vaccine molecule.

### 3.2 Design of TG_200 mRNA construct and its expression in 293T cells

The construct was designed based on the TG*_*200 coding sequence and sequences were added to make it more complete and stable. The UTRs contributed to mRNA stability and translation regulation, which was critical for optimal protein expression. We transcribed mRNA from the T7 RNA polymerase promoter site upstream of the 5′ UTR *in vitro*. The modified mRNA was synthesized from a linearized DNA template *in vitro*.

**Figure 3:**
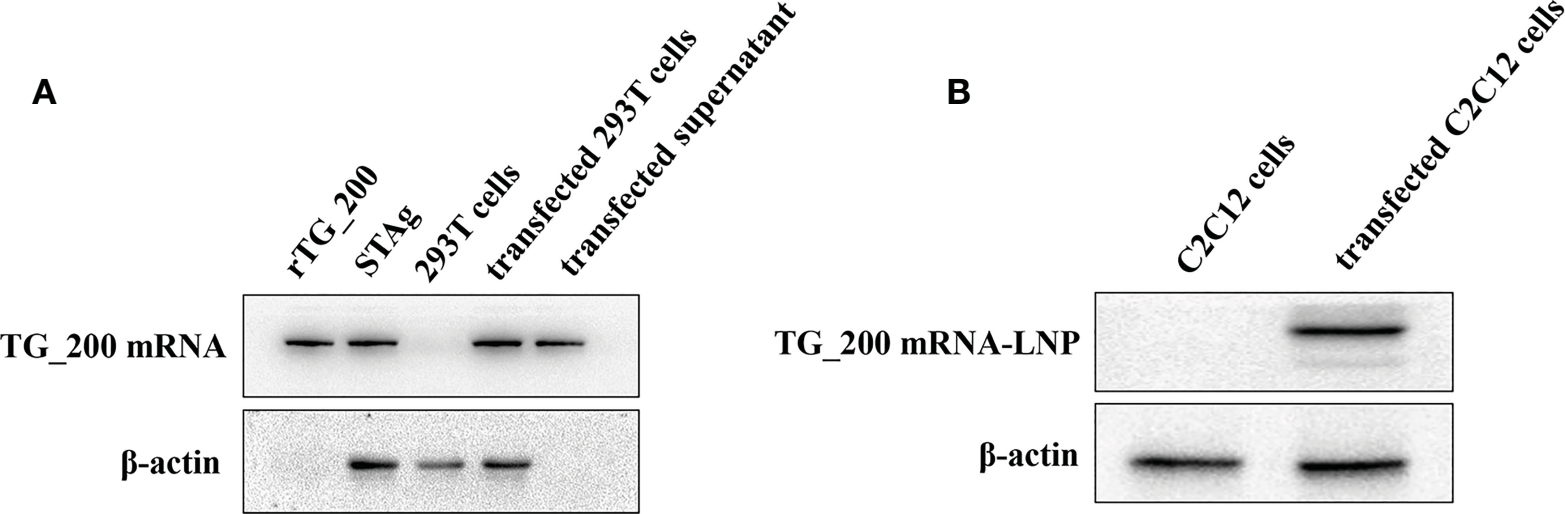
Expression of TG_200 mRNA in 293T cells and TG_200 mRNA-LNP in C2C12 cells. **(A)** Western blotting detection of the expression of TG_200 protein in rTG_200, STAg, 293T cell lysates and TG_200 mRNA transfected 293T cells supernatant. rTG_200: the recombinant pET28a-TG_200 acting as a positive control; STAg: soluble antigen of *T. gondii*; 293T cells; transfected 293T cells: TG_200 mRNA transfected 293T cells; transfected supernatant: TG_200 mRNA transfected 293T cell supernatant. **(B)** Western blotting detection of TG_200 mRNA-LNP in C2C12 cells. C2C12 cells: C2C12 cell lysates; transfected C2C12 cells: TG_200 mRNA-LNP transfected C2C12 cells lysates.

TG_200 mRNA construct was transfected in 293T cells, and its expression was analyzed by WB (**Figure 3A**). In WB, the rabbit anti-TG_200 polyclonal antibody detected single specific protein bands in 293T cells transfected with rTG_200, STAg, TG_200 mRNA, and supernatant. The first band was detected, indicating that the antibody was correct and viable. The presence of a band in the STAg sample (second band) indicated that the TG_200 protein is soluble. No bands were detected in untransfected 293T cells. Additionally, we detected bands in the supernatant of transfected cells; however, no internal control bands were detected in the supernatant sample, indicating that the sample was not contaminated by cells and that the TG_200 protein was a secreted protein.

### 3.3 TG_200 mRNA-LNP delivery and expression in C2C12 cells

C2C12 cells were selected for transfection to ensure that TG_200 mRNA-LNP could be expressed in myocytes for subsequent intramuscular injection. In **Figure 3B**, TG_200 mRNA-LNP levels were measured indirectly by WB detection of TG_200. In C2C12 cells transfected with TG_200 mRNA-LNP, a single specific protein band was detected, and there was no band in the untransfected cells. The result indicated that the TG_200 mRNA-LNP was successfully transfected and expressed. Assessment of the encapsulation efficiency using Quant-iT™ RiboGreen™ RNA Reagent and Kit to measure the concentration of free and total RNA in the post-encapsulation solution showed that the encapsulation efficiency obtained was about 95.67%.

### 3.4 Humoral immune responses induced by vaccination with TG_200 mRNA-LNP

**Figure 4:**
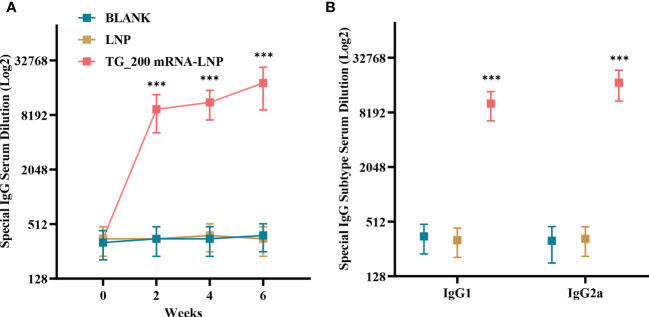
Detection of *T. gondii* specific humoral immune responses induced by TG_200 mRNA-LNP immunization. **(A)** Serum from vaccinated mice was isolated at 0, 2, 4, and 6 weeks after vaccination and analyzed for IgG neutralization of serially diluted serum samples, n = 8/group. ***P< 0.001. **(B)** Serum from vaccinated mice was isolated 2 weeks after the boost and analyzed for IgG1 and IgG2a neutralization of serially diluted serum samples, n = 8/group. ***P< 0.001. Data are presented as the means ± SD.

ELISA was conducted to detect serum IgG, IgG2a, and IgG1. A higher IgG titer was detected in the serum of mice immunized with TG_200 mRNA-LNP than in the BLANK and LNP groups (**Figure 4A**). Significant differences in IgG levels began to appear between the TG_200 mRNA-LNP immunized group and the control at two weeks after the first immunization (*P*< 0.001). There was no significant difference in antibody levels between the groups before immunization, and antibody titer increased with the number of immunizations (until week 6). No significant difference was observed between the BLANK and LNP groups from week 0 to week 6 (*P* > 0.05). Immunization with the TG_200 mRNA-LNP vaccine increased IgG antibody levels in mice, inducing corresponding humoral immunity. **Figure 4B** shows that compared with those in the control group, IgG1 and IgG2a in the TG_200 mRNA-LNP immunized group were significantly elevated (*P*< 0.001). The IgG2a/IgG1 ratio in the TG_200 mRNA-LNP immunized group was higher than that in the controls, indicating a dominant T helper type 1 (Th1-type) cellular immune response induced by the TG_200 mRNA-LNP vaccine. Increased antibody levels following immunization suggested increased protection against *T. gondii* infection.

### 3.5 Analysis of the cellular immune response

**Figure 5:**
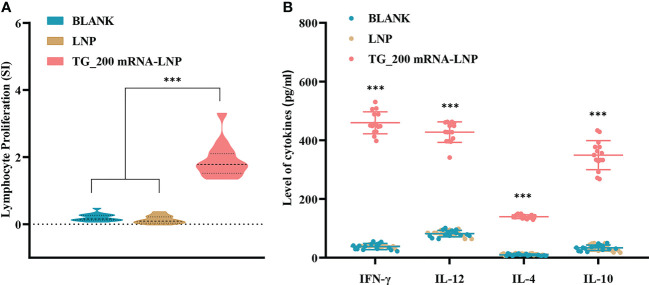
Splenocyte proliferation and cytokine production levels after TG_200 mRNA-LNP immunization. **(A)** Proliferative responses of splenocytes of BALB/c mice immunized with LNP, TG_200 mRNA-LNP, or the BLANK control, n = 5/group, ***P< 0.001. **(B)** Cytokine production by splenocytes of mice immunized with LNP, TG_200 mRNA-LNP, or the BLANK control. The vertical axis is the cytokine concentration (pg/mL). The horizontal axis shows IFN-γ, IL-12, IL-4, and IL-10, n = 5/group, ***P< 0.001. Data are shown as the means ± SD of three independent experiments.

Immune cell responses to TG_200 mRNA-LNP were evaluated using spleen cell suspensions prepared from each mice two weeks after the last immunization. **Figure 5A** shows that compared with the medium and LNP groups, splenocytes from mice immunized with TG_200 mRNA-LNP showed an enhanced proliferative response (*P<* 0.001). There was no significant difference between the control groups (*P* > 0.05). Lymphocyte proliferation and differentiation are essential stages in the immune response process, and the lymphocyte proliferation test is the classical test to detect cellular immune function. A significant increase in lymphocyte proliferation indicated the production of specific cellular immunity against the TG_200 mRNA-LNP molecule.

IL-12 can induce IFN-γ release. In turn, IFN-γ plays an essential immunomodulatory role as a cytokine secreted by immunoreactive cells in the induction of immunity against infection, including the activation of natural killer cells (NK), CTLs, and phagocytes. IFN-γ and IL-12 are Th1 cytokines, and IL-4 and IL-10 are Th2 cytokines. Th2 cytokines regulate the pathological inflammatory response of Th1 cytokines due to strong immunity during the fight against infection to balance the stability of the normal immune system. Therefore in the current experiment, Th1 and Th2 cytokines were tested separately. Levels of IL-12, IFN-γ, IL-10, and IL-4 were measured in the spleen cell supernatant from spleens taken from mice after the last two weeks of immunization. **Figure 5B** shows that compared with those in the BLANK and LNP groups, higher levels of IFN-γ (459.5 ± 37.52), IL-12 (427.7 ± 34.91), IL-4 (139.7 ± 6.363), and IL-10 (349.2 ± 49.46) were detected in TG_200 immunized mice (*P<* 0.001). The result indicated that a mixed Th1 and Th2 immune response was produced in mice immunized with TG_200 mRNA-LNP. Thus, an effective immune response was produced against *T. gondii* infection.

### 3.6 High CTL activity detected in mice immunized with TG_200 mRNA-LNP

**Figure 6:**
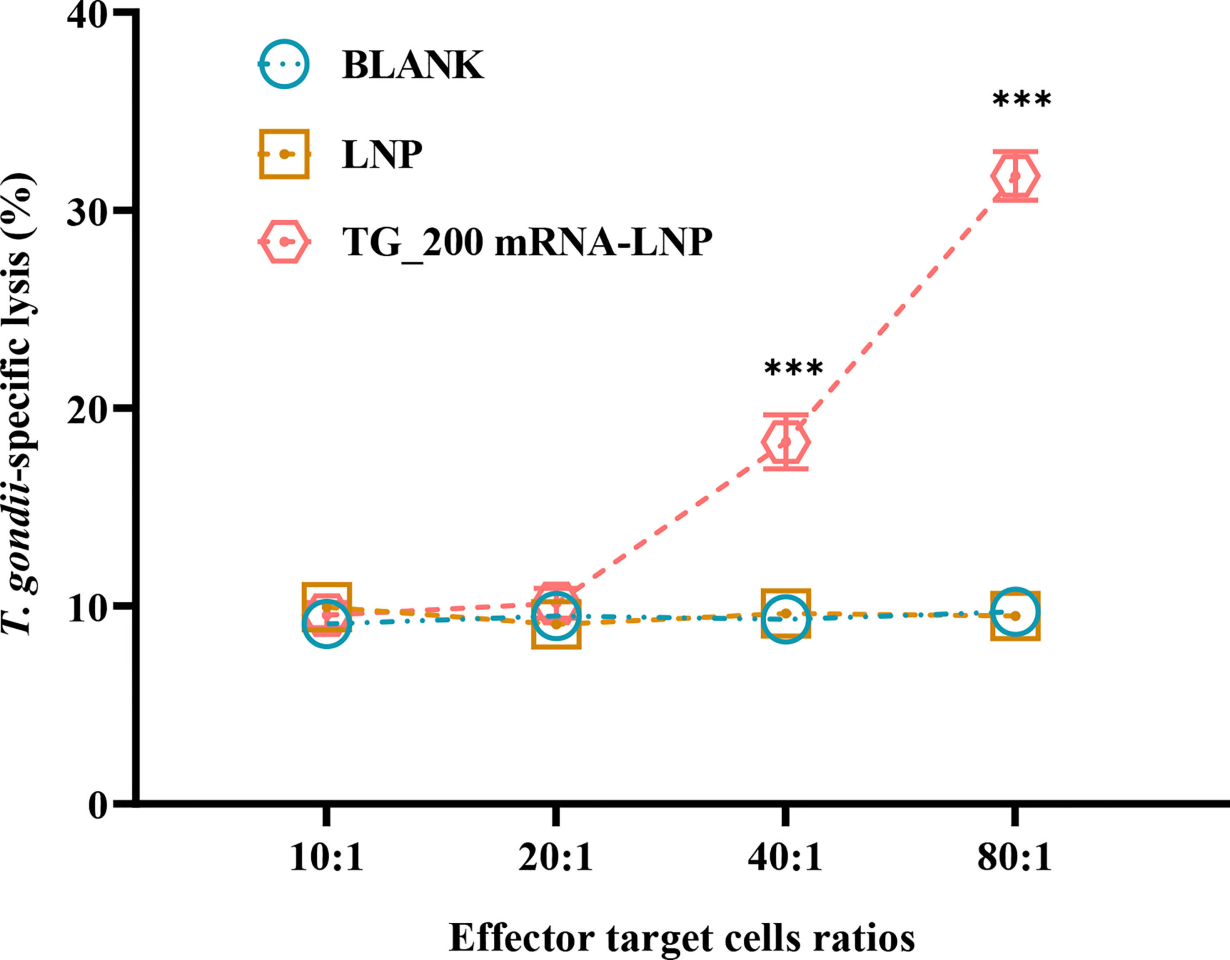
CTL activities of spleen lymphocytes in TG_200 mRNA-LNP immunized mice. The vertical axis shows *T. gondii*-specific lysis as a percentage of the total possible lysis (%). The horizontal axis is the ratio of effector cells to target cells. n = 5/group, ***P< 0.001.

CTLs plays a vital role in the fight against intracellular pathogen infection. The killing activity of CTLs is antigen-specific and is limited by MHC class I molecules. It requires the same MHC allele product for initial sensitization and resensitization or the same antigenic peptide-binding common motif. One CTL can kill multiple target cells in succession and is highly efficient. Therefore, we evaluated the specific cellular immunity induced by the vaccine by measuring the cytotoxic lysis activity of splenic lymphocytes. **Figure 6** shows that the CTL activity of splenocytes from mice immunized with TG_200 mRNA-LNP increased progressively as the effector-to-target cell ratio increased. A significant difference was observed when the effector-target cell ratio was 40:1 and above compared with that in the controls (*P*< 0.001). When the ratio was 80:1, CTL activity reached higher values (*T. gondii*-specific lysis accounted for more than 30% of total possible lysis). Splenic lymphocyte CTL activity between the control groups was not significantly different (*P* > 0.05). The result showed that pathogen-specific CTL responses were induced and produced effective immune responses to intracellular pathogens.

### 3.7 Flow cytometry analysis

**Figure 7:**
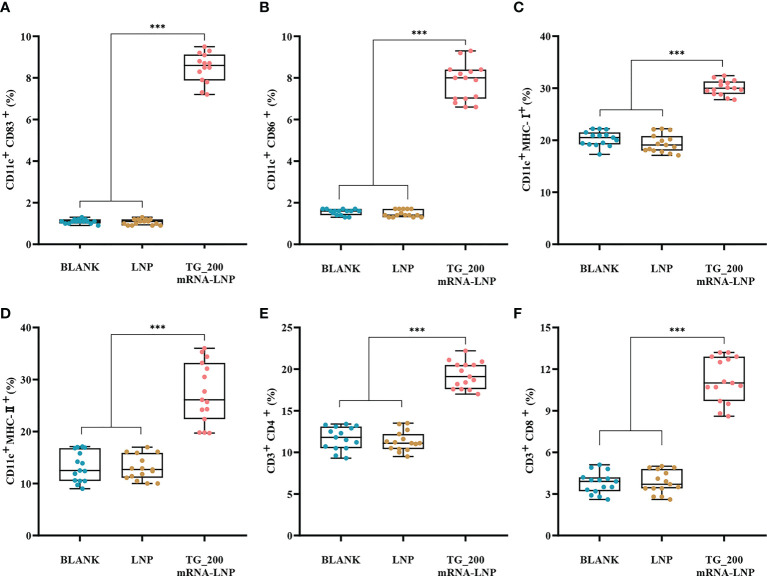
Flow cytometry analysis of dendritic cells (DCs) and T lymphocytes in immunized BALB/c mice. **(A)** The box graph shows the percentages of CD11c^+^ CD83^+^ DCs in mice splenocytes. **(B)** The box graph shows the percentages of CD11c^+^ CD86^+^ DCs in mice splenocytes. **(C)** The box graph shows the percentages of CD11c^+^ MHC-I^+^ DCs in mice splenocytes. **(D)** The box graph shows the percentages of CD11c^+^ MHC-II^+^ DCs in mice splenocytes. **(E)** The box graph shows the percentages of CD3^+^ CD4^+^ T lymphocytes. **(F)** The box graph shows the percentages of CD3^+^ CD8^+^ T lymphocytes. n = 5/group, ***P< 0.001. The data are shown as the means ± SD of three independent experiments.

The gathering of inflammatory mononuclear cells at the site of infection is also essential for controlling *T. gondii* infection. We determined whether DC differentiation was activated by detecting elevated expression levels of CD83, CD86, MHC-II, and MHC-I on the surface of DCs in mice after immunization with TG_200 mRNA-LNP. CD4^+^ T cells play an important role in the acute phase of infection. CD8^+^ T cells and CD4^+^ T cells were the main cells controlling parasitic IFN-γ production in chronically infected mice. Therefore, flow cytometry was performed to determine whether TG_200 mRNA-LNP vaccination activated DC cells and to analyze the MHC molecules in DCs and the proportion of CD4^+^ and CD8^+^ T lymphocytes subsets. **Figures 7A, B** show higher DC surface expression of CD83^+^ and CD86^+^ in the TG_200 mRNA-LNP group than in the control groups (*P*< 0.001). This indicated that DC cells are efficiently activated and differentiated. **Figures 7C, D** show that after TG_200 mRNA-LNP immunization, there was a higher level of MHC-I and MHC-II molecules than those in the controls (*P*< 0.001). In addition, **Figures 7E**, F show that mice immunized with TG_200 mRNA-LNP had a significant increase in the percentage of CD8^+^ and CD4^+^ T cells compared with those in the control group (*P*< 0.001). There were no significant differences between the control groups (*P* > 0.05). The results showed that the vaccine activated CD4^+^ T cells and CD8^+^ T cells, eliciting an effective cellular immune response. Combined with the CTL results, this suggested that the vaccine induced an effective and specific cellular immune response, which is critical for killing intracellular parasites.

### 3.8 The expression of cytokine-related transcription factors

**Figure 8:**
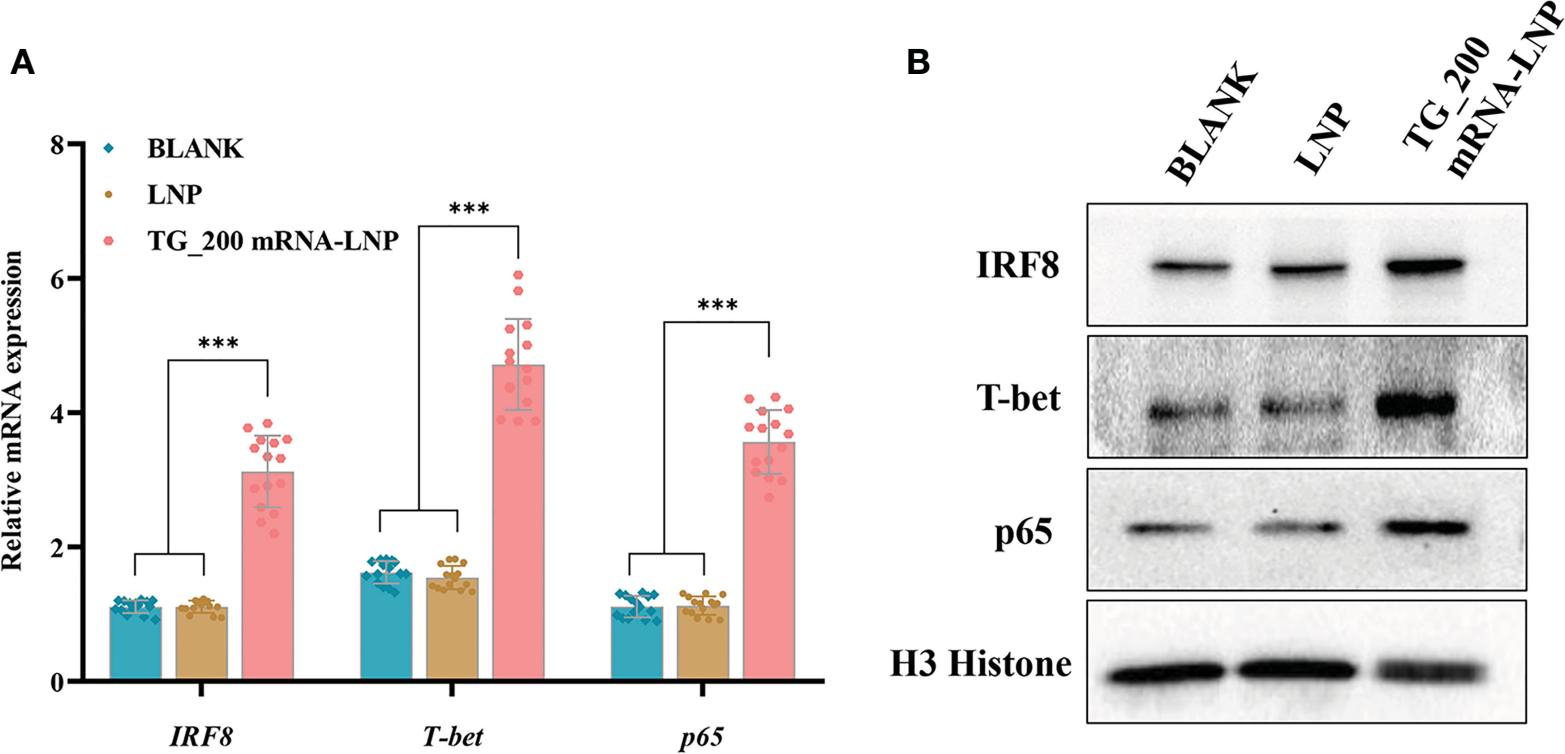
The mRNA and protein expression levels of IRF8, T-bet, and p65 in mice splenocytes. **(A)** mRNA levels of *Irf8*, *Tbet*, and *p65*. n = 15/group, ***P< 0.001. **(B)** Protein levels of IRF8, T-bet, and p65, BLANK: Splenocytes from untreated mice; LNP: Splenocytes lysates from the LNP injected mice; TG_200 mRNA-LNP: Splenocytes lysates from mice injected with TG_200 mRNA-LNP.

The p65 molecule is a member of the NF-κB family, and NF-κB transcription factors are essential in the fight against *T. gondii* infection. Interferon regulatory factors (IRFs) can activate DCs to produce IFNs and cytokines. T-bet can specifically promote Th0 differentiation into Th1 cells, which is important in intracellular pathogen infection. qRT-PCR was used to detect the mRNA expression of transcription factors *Irf8*, *p65*, and *Tbet*. Compared with those in the control groups, *Irf8*, *p65*, and *Tbet* mRNA levels were significantly higher in the TG_200 mRNA-LNP immunization group (*P*< 0.001) (**Figure 8A**). There was no significant difference between the LNP and the BLANK groups (*P* > 0.05). Furthermore, WB showed that the levels of the IRF8, p65, and T-bet were higher in the TG_200 mRNA-LNP group compared with those in the two control groups (**Figure 8B**). At the same time, the expression of internal control gene (H3) was consistent among the three groups of samples. The WB results were consistent with the qRT-PCR results, indicating that TG_200 mRNA-LNP immunization can effectively activate the NF-κB pathway and DC cells, in addition to promoting the specific cellular immunity dominated by a Th1-type immune response.

### 3.9 Immunoprotection against lethal challenge and transfer studies

**Figure 9:**
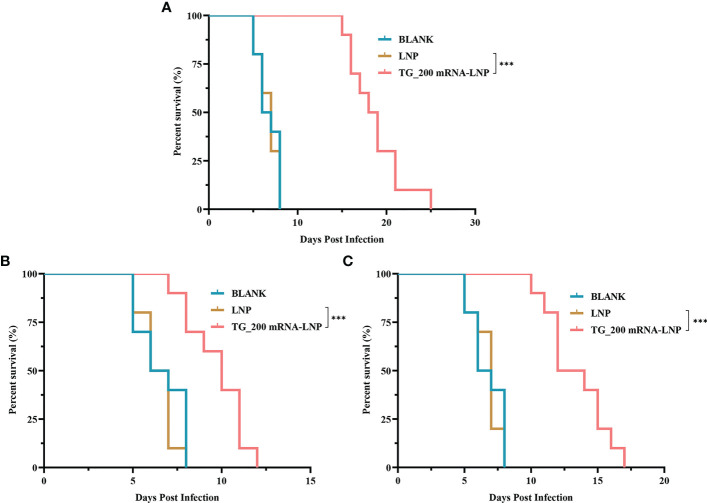
The survival rate of mice immunized with TG_200 mRNA-LNP and adoptively transferred serum or splenocytes from vaccinated mice. **(A)** The survival rate of BALB/c mice immunized with TG_200 mRNA-LNP, LNP and BLANK group and challenged with 1×10^2^ *T. gondii* RH tachyzoites two weeks after the last immunization, n = 10/group. **(B)** The survival rate of mice receiving adoptively transferred serum (daily for five days) from mice vaccinated with TG_200 mRNA-LNP, LNP and BLANK group and challenged with 1×10^2^ *T. gondii* RH tachyzoites, n = 10/group. **(C)** The survival rate of mice receiving adoptively transferred splenocytes (5×10^7^) from mice vaccinated with TG_200 mRNA-LNP, LNP and BLANK group and challenged with 1×10^2^ *T. gondii* RH tachyzoites, n = 10/group. ***P< 0.001.

Mice were challenged with *T. gondii* RH strain to assess the protective effect of the TG_200 mRNA-LNP vaccine. The survival curves in **Figure 9A** show that the survival time of TG_200 mRNA-LNP immunized mice was prolonged after challenge with 1 × 10^2^ *T. gondii* RH compared with that of the mice that received LNP or BLANK controls (19.27 ± 3.438 days, *P*< 0.001).

Although sera from TG_200 mRNA-LNP vaccinated mice have antibodies that inhibit *T. gondii* reproduction, the protection offered by the immunized sera is limited. **Figure 9B** shows that the survival of mice treated with adoptively transferred serum from vaccinated mice was increased after challenge with 1 × 10^2^ *T. gondii* RH compared with that of the mice treated with serum from control mice (9.70 ± 1.64 days, *P<* 0.001).

Splenocytes (5 × 10^7^) from TG_200 mRNA-LNP inoculated mice were transferred successively to mice, and the survival of the successively transferred mice was monitored after challenge with 1 × 10^2^ *T. gondii* RH tachyzoites. **Figure 9C** shows that compared with the mice receiving LNP or BLANK controls, the survival time of splenocyte-immunized mice was prolonged (13.40 ± 2.32 days, *P*< 0.001).

## 4 Discussion

*T. gondii* can infect all warm-blooded animals. Human infections are acquired from contaminated food or water, and it is estimated that this parasite persists chronically in 25–30% of the global human population. Zoonotic parasitic diseases cause serious public health problems and threaten economies and productivity. To date, combination therapy with pyrimethamine and sulfadiazine has been used to treat toxoplasmosis; however, these drugs have unavoidable side effects. Therefore, researchers have sought to develop *T. gondii* vaccines; however, although there are many types under research, few vaccines offer complete protection and there are problems related to their safety and efficacy. Therefore, a safe and effective *T. gondii* vaccine is urgently required. Compared with conventional vaccines, mRNA vaccines have the potential for high potency, rapid development, safe delivery, and low-cost manufacturing (23). Furthermore, successful mRNA vaccines have been clinically developed and licensed in a short time (for example, the COVID-19 mRNA vaccine) (24, 25). There are also many mRNA vaccines developed to prevent parasitic diseases, for example, the mRNA vaccine protection against pre-erythrocytic malaria, which shows good protection rates (26). In anti-*T. gondii* mRNA vaccine research, only one article has been published, which described a significantly enhanced protective effect of the vaccine (27). We studied the possibility of using new *T. gondii* molecule, TG_200, as an mRNA vaccine molecule to prevent toxoplasmosis in mice. The results showed that mice immunized with TG_200 mRNA-LNP generated cellular and humoral immunity, which prolonged the survival of mice challenged with *T. gondii*.

There are many *T. gondii* vaccine research platforms, and in comparison, mRNA vaccines theoretically avoid the risk of genetic recombination and are relatively safe, with little risk of misfolding recombinant proteins. In addition, independent cell expansion shortens the production time and is more conducive to preventing epidemic diseases (28). In this study, the survival time of the mice was prolonged during *T. gondii* challenge and appeared to be more effective compared with some other platform vaccines: epitope vaccines, recombinant protein vaccines, DNA vaccines and carbohydrate-based vaccines (29–33). However, comparisons between different laboratories are difficult due to the lack of standardized protocols for *T. gondii* vaccine studies (28). There are variations in immunization routes, animal models, dosages, and vaccine production processes.

Secretory proteins play an important role in host-pathogen interactions (19); therefore, the search for vaccine candidate molecules on the *T. gondii* database (ToxoDB, http://ToxoDB.org) focused on predicting *T. gondii* secretory proteins. After screening for genome-wide secreted proteins containing transmembrane structural domains and signal peptides, further T/B cell epitope prediction of the screened candidate molecules revealed that the TG_200 molecule had better predicted scores of T/B cell epitopes. Epitope prediction plays an important role in the analysis of peptide immunogenicity and plasmid vaccine construction (20, 21). The epitopes of many *T. gondii* vaccine molecules, such as MIC4 (34), SAG1 (35), and GRA24 (17), have been predicted using bioinformatics. Besides, adaptive immune responses are usually mediated by T and B cells to provide immune protection. In the present study, bioinformatic analysis of TG_200 predicted its potential as a vaccine. The DNASTAR software suggest that TG_200 has good surface probability, hydrophilicity, a high antigenic index, and more variable regions. Moreover, the T-cell epitopes of TG_200 were predicted to be capable of binding strongly to MHC II molecules. Bioinformatic analysis also showed that the percentile IC50 value was lower than that of SAG1 and the linear B-cell epitope score of the TG_200 peptide was higher, suggesting that TG_200 has promising potential for vaccine production.

Unfortunately, mRNA vaccines are easily degraded *in vivo* and have a short intracellular half-life. In addition, it is almost impossible for naked mRNA to cross the cell membrane successfully because of its physicochemical properties, such as the large size of the mRNA molecule and its dense negative charge (23). Thus, effective and appropriate delivery systems are needed to induce robust humoral and cellular immunity and prevent antigen degradation in mRNA vaccines (36). Many materials have been developed to deliver mRNA, including lipids, polymers, protein derivatives, and lipid-like materials (23, 37–40). Among them, as one of the most attractive and widely used delivery tools for mRNA, LNPs have been thoroughly investigated and successfully applied in the clinic (41). BNT162b21 (42) and mRNA-1273 (43) are two currently approved anti-coronavirus disease 2019 (COVID-19) vaccines that use LNPs to deliver antigenic mRNA. LNPs have been evaluated as effective and safe, representing a mature technology. Therefore, LNPs were chosen as the TG_200 mRNA vaccine delivery system. The encapsulation efficiency of the TG_200 mRNA vaccine in LNPs was about 95.67%. 293T cells were selected to test the expression of TG_200 mRNA *in vitro*. In clinical practice, most vaccines are administered by intramuscular injection and myocytes dominate muscle tissue. Therefore, C2C12 cells were chosen to detect the expression of TG_200 mRNA-LNP in muscle cells. The results show that the TG_200 protein is correctly expressed in cells (293T and C2C12) and is a secreted protein. And the protein might be taken up by cells and presented by the MHC-II molecule, which is mainly involved in the presentation of exogenous antigens and is usually expressed on antigen presenting cells (APCs) such as monocytes-macrophages, B cells, and DCs.

In the anti-*T. gondii* infection response, humoral immunity plays a critical role. Moreover, the B-cell response is important in infection by toxoplasmosis (44). Anti-*T. gondii* IgG antibodies control *T. gondii* infection by inhibiting *T. gondii* replication. IgG allows macrophages to kill intracellular parasites through the antibody-dependent cell-mediated cytotoxicity (ADCC) pathway by promoting parasite attachment to immune cells (45). In addition, the Th1 immune response plays an important role in resistance to *T. gondii* infection. One indicator of the Th1 immune response is the IgG2a level, while IgG1 is an indicator of the Th2 immune response. Levels of IgG2a and IgG1 antibodies were significantly increased in the TG_200 mRNA-LNP group. The IgG2a/IgG1 ratio also increased, indicating that TG_200 mRNA-LNP induced a mixed humoral Th1 and Th2 (Th1 dominated) immune response. This response is associated with increased protection against infection, which is similar to DNA vaccines, such as that of TgDOC2C (46), GRA7 (47), GRA24 (17), and other antigens.

The production of Th1-type cytokines IFN-γ and IL-12 mediates Th1 cell activity against intracellular pathogens (including *T. gondii*), representing the main anti-infection effector (48). IL-12 and IFN-γ expression levels in the splenocyte culture supernatant of TG_200 mRNA-LNP immunized mice were significantly higher than those of the control group (*P<* 0.001). IL-12 can induces IFN-γ release and Th1 cell differentiation, and thus control *T. gondii* infection. Blocking IL-12 leads to a weak Th1 immune response and promotes *T. gondii* growth and transmission (49). IFN-γ is secreted by NK cells through a toll-like receptor-myeloid differentiation factor 88 (MyD88)-dependent signaling pathway that is synergistically triggered by *T. gondii* antigen and IL-12 (50). IFN-γ is important for limiting the growth of tachyzoites in the early stages of infection, inhibiting the reactivation of dormant *T. gondii* cysts (51), inhibiting the growth and reproduction of *T. gondii* through the degradation of amino acids (tryptophan, L-arginine) essential for growth, and by inducing immune-related GTPases (IRGs), which can destroy vacuoles and eliminate parasites by lysosome-mediated degradation (48, 52). Immunization with the vaccine also significantly induced Th2-type cytokines, such as IL-10 and IL-4 (*P*< 0.001). Immunopathology can also be fatal during acute *T. gondii* infection, and the accompanying IL-10 and IL-4 responses can prevent an intense inflammatory response by suppressing systemic Th1 cytokine production and preventing the development of immunopathology (48). Increased inflammatory cell infiltration and intense necrosis were found in KO IL-10 mice, and their mortality was associated with enhanced liver pathology (53). IFN-γ performs an essential role in the early stages of *T. gondii* infection, and IL-4 is generally antagonistic to IFN-γ. During acute and chronic infection with *T. gondii*, IL-4 is necessary to suppress severe immunopathology. IL-4 has is also important in the late stages of infection, in which it can promote IFN-γ production. KO IL-4 mice lead to increased susceptibility to severe *T. gondii* encephalitis, of impaired IFN-γ production (52). In our experiments, Th1 and Th2 immune cells were stimulated to produce effective protection, prevent severe immunopathology caused by killing pathogens, and control host immune responses. There are some vaccine candidates whose results are consistent with our results: GRA24-based DNA Vaccine (17), MYR1-based DNA Vaccine (54). However, some vaccine candidates showed that they only produce a Th1-type immune response to control pathogenic infections: the GRA7-based DNA vaccines (47), and TgMIC5 and TgMIC16-based DNA vaccines (29).

DCs can produce type I cytokines and IFN, which are important in innate immunity. An essential checkpoint, CD83, is often expressed on the surface of mature DCs. It plays an important role in the regulation of immunity and the induction of inflammation regression (55). CD86 binds to CD28 molecules to provide co-stimulatory signals from T cells, thereby lowering their naive activation threshold (56). In the current study, significant enhancement of CD83 and CD86 molecules was observed in animals immunized with TG_200 mRNA-LNP vaccine, suggesting the promotion of DC maturation and increased expression of co-stimulatory molecules. Previous studies indicated that *T. gondii* promotes DC maturation by detecting its marker molecules, such as CD86 (57). Mature DCs can produce MHC-II molecules that present antigens and activate CD4^+^ T cells. This study observed significantly high levels of MHC-II molecules in mice immunized with TG_200 mRNA-LNP, suggesting an important role in antigen presentation. MHC-I molecules are often expressed in nucleated cells, and are essential for endogenous antigen presentation, leading to CD8^+^ T cell activation (58). The present study indicated that antigen presentation is induced in a mainly MHC-II, and slightly MHC-I, dependent manner. This suggests that TG_200 mRNA-LNP promotes DC maturation. Resistance to *T. gondii* is dominated by innate immunity based on NK cells and adaptive immunity based on CD4^+^ T lymphocytes. Activated CD4^+^ T lymphocytes can activate macrophages, and then produce cytokines that are recruited to the infection site (59). In addition, activated CD8^+^ T cells differentiate into CTLs, exhibiting immunotoxicity against the parasite (60). In the present study, a significant increase in CD4^+^ and CD8^+^ T lymphocytes compared with that in the control group suggested that TG_200 mRNA-LNP plays an important role in the induction of CD4^+^ and CD8^+^ T lymphocytes against toxoplasmosis. Studies of nano vaccines ribosomal P2 (61) and GRA7 (17) of *T. gondii* showed a significant increase in CD4^+^ and CD8^+^ T lymphocytes, similar to this study. In addition, mice immunized with TG_200 mRNA-LNP were found to have higher CTL activity than the controls. Our results indicated that TG_200 mRNA-LNP induced a pathogen-specific CTL response and an effective immune response to intracellular pathogens. These results are similar to those reported for the GRA24-based DNA Vaccine (17) and the MYR1-based DNA Vaccine (21).

IFN-γ can stimulate innate immune cells to upregulate immunity-related GTPases, such as p65 guanylate-binding protein (Gbp) family genes. p65, also known as RelA, is one of the five components of NF-κB. NF-κB is a transcription factor and enhancer of activated B cells. Host NF-κB plays a key role in blocking *T. gondii*-mediated apoptosis, and this inhibition is lost in p65 KO cells (62). This indicated that p65 is an important component of NF-κB, which plays an important role in the control of *T. gondii* infection. IRF family proteins activate DCs to produce IFN and cytokines (IL-10 and IL-12) (63). IL-12 is also critical to the control of *T. gondii* infection. *T. gondii* profilin can induce IL-12 through MyD88 and IRF8-dependent signaling pathways, and IRF8 is a critical transcription factor that regulates IL-12 activation of downstream TLR11 and MYD88 expression. Failure to upregulate IRF8 in DCs leads to acute susceptibility to *T. gondii* infection (62). T-bet plays a key role in the Th1/Th2 transition. It can specifically regulate Th0 differentiation and is selectively expressed in Th1 cells. Innate IFN-γ, which is T-bet dependent, is critical for the induction of IRF8. Thus, IRF8, T-bet, and p65 molecules have a crucial role in the immunoprotective response to *T. gondii*; therefore, we assessed their expression levels after vaccination. The spleen lymphocytes of TG_200 mRNA-LNP immunized mice showed a significant increase in IRF8, T-bet, and p65 expression. The results suggested that TG_200 mRNA-LNP can induce an immune response against *T. gondii* infection. The promotion of IFN-γ production is also mediated through activation of the IRF8 pathway, T-bet, and NF-κB pathways (64, 65), which is consistent with our results.

After *T. gondii* challenge, the survival of vaccinated mice was assessed. Survival time is a direct and credible indicator of the safety and efficacy of candidate *T. gondii* vaccines. We evaluated the survival of vaccinated mice by intraperitoneal infection with the RH strain of *T. gondii* two weeks after the last immunization with TG_200 mRNA-LNP. Compared with that of the controls, the survival time of the mice immunized with TG_200 mRNA-LNP increased significantly (19.27 ± 3.438 days, *P*< 0.001), although after infection, the mice did not survive. Thus, after infection with highly virulent *T. gondii*, the TG_200 mRNA-LNP vaccine induces better partial protection, although it does not induce complete protection; however, its induced survival time was better than some other *T. gondii* vaccines. GRA14-based DNA vaccination induced high levels of IFN-γ, total IgG and IgG2a, and Th1-type dominant humoral cellular immune responses. However, although BALB/c mice challenged with a lethal dose of RH strain tachyzoites had the longest survival time, it was only up to 17 days (66). The survival time of mice immunized with recombinant calcium-dependent protein kinases (rTgCDPK1) was significantly prolonged (14.90 ± 2.89 days) with a higher level of IgG antibodies and significantly increased levels of Th1-type cytokines (67). The survival time of BALB/c mice injected with pVAX-rhoptry proteins 21 (TgROP21) was also significantly prolonged (13.50 ± 1.65 days) (68). Survival time was also analyzed using immunized mice sera and splenocytes to assess the protective effect of the vaccine. Herein, after challenge with 1 × 10^2^ RH tachyzoites, the mice survival time was prolonged when accepting immunized mice splenocytes (13.40 ± 2.32 days). After receiving transfer of CD8^+^ or CD4^+^ splenocytes from Hsp70-immunized mice, the *T. gondii* load was limited in the acute and chronic phases of toxoplasmosis (69). Compared with the control group, adoptive transfer of the splenocytes of KO tyrosine kinase (KO tkl1) immunized mice enhanced the survival time (70). The results indicated that CD8^+^ and CD4^+^ CTL are functional effectors of host resistance to *T. gondii*. Successive splenocyte transfers might better control disease recurrence and are important to develop better immunotherapies against *T. gondii*. The mice challenge with 1×10^2^ RH tachyzoites also showed a prolonged survival time when accepting immunized mice serum daily for five days (9.70 ± 1.64 days, *P*< 0.001). A study also indicated that the survival time of mice accepting serum from KO tkll immunized mice was increased compared with the controls (70). For better immune protection against *T. gondii* infection, appropriate adjuvants might be required or the vaccines might be administered in combination with other important molecules of *T. gondii.* It has been shown that the protection produced by multiple proteins or epitopes is better than that by single proteins or epitopes (29, 71, 72).

In this study, a mRNA vaccine based on a novel *T. gondii* molecule-TG_200 was successfully constructed and BABL/C mice were used as the experimental model to assess immune protection through humoral, cellular immunity, and survival rates induced by the vaccine. The TG_200 mRNA-LNP vaccine elicits high levels of IL-12, IFN-γ, IL-10, IL-4, and increased IgG2a/IgG1 ratios. The results indicate that the novel vaccine can elicit Th1 and Th2 immune responses, with Th1 immunity being predominant. Furthermore, under immunization by the TG_200 mRNA-LNP vaccine, the ability to increase lymphocyte proliferation was significantly enhanced. After *T. gondii* challenge, mice immunized with the TG_200 mRNA-LNP vaccine had a significantly prolonged survival time. After receiving serum and splenocytes from immunized mice, the survival time was also significantly prolonged. Thus, TG_200 mRNA-LNP might be a promising vaccine against *T. gondii* infection after adjustment of the adjuvant or combination with other *T. gondii* critical molecules.

## Data availability statement

The raw data supporting the conclusions of this article will be made available by the authors, without undue reservation.

## Ethics statement

The animal study was reviewed and approved by Chinese legislation on the use and care of laboratory animals (GB/T35823-2018). Hangzhou Medical College Institutional Animal Care and Use Committee (No: 2021-152).

## Author contributions

BZ and SHL developed the study protocol. YZ, DL, YS, and SYL did the experiments. YZ, DL, and YS analyzed the data and wrote the paper. BZ and SHL revised the paper. All authors contributed to the article and approved the submitted version.
