# Supplementary material for: Immunization with a novel mRNA vaccine, TGGT1_216200 mRNA-LNP, prolongs survival time in BALB/c mice against acute toxoplasmosis
Source: Front Immunol. 2023 Apr 14;14:1161507. doi: 10.3389/fimmu.2023.1161507 (PMC10140528; doi:10.3389/fimmu.2023.1161507)
Supplement: Supplementary file 1 [file DataSheet_1.docx]

**AATAATACGACTCACTATAGGGG**ATATTTGACCGCGAGAACTCGCAGTATTCGCTACCGTGTGGAGCCTCGATATATCTCTGGTTCTTCGAGCAAATACTCCAGCCCGGCACTCTCCGAAACTCTTATGTACACTTGGAGGTCAAGTGCCGCGACTGAAGACGTGGTTCCATTCATTCTGCAGCTACGGTTCCTGACTGGCATCGCTCGGTTTGTCGTCCGACTGGCGGGCGCCATTGGAACGAGGCGTCGCTTGCAAAATAACGCCCCCGCAGCCCGGAACAGCTATAACATTAAGAGCACTTTTTTCAGCGGGGTCCGCCGATGGCGTTAACCATCTGTTGGCATGTCACGATGACGCAGACGTCGTTTTCTTTCCGTGTCGTTTGGGTTGTTTGGGGTTCGAGGCATGGGCAGACACGCGAAGCCCCTGCCTCCACCGAAACGGATCGGTACGCCGGACTTGAAGAAAAGCGCGGCTCGACGGGATCACCGAAACCCGCCAGTCGCCAATCCGTGAAGAGGACCCGGGCGCCTACACGCTGTCGGCCGTTCACCCTTCTTAGTCTTGGACGGTGGAACCGTGTACACACCAGGTCCAGTACTCACGTGACCTCGCGTGACCTCTTCGCCGATTCGCTTATGGTATATGAACACCCTAGGCGAGGCGTACTTGTCTGGCCCCCCGTTTGTGCGTCTGCAAGTTTTGTGCCAAGAGAGGTCGCTGGTTTGTTGTCGCGGTTGGATCTTCATGGAATTGTGTCCGATTTTTTCCCTACAGGGCTGAGAAGAATTGTGGGAGGAGGCCGAAGCAACGAACCTCCGCATCGGTCGCACCGGCGATGTTTTCCGCCGCCGCTCCATTCTCACCCATTGTTGCTCTCTGGCCTGGTAGGATTCCGCAGTGACTCTCGGGGAGTTTGTGCCTGTCACCTGTACAAGTCGACGGATGCCTGTATTTTCGAAAAACGACGTGTCGAGCGGAGAGAAAGCAGGGAAACTGGTAAGAAGCGTCGGAGGTGCACGGCCAGTAGCGCCTGAGAAGAGACTGGTCGAGACTGGTCCGGTGTCGCGCCGTCCGAGGGCCGAAACGGGGCCGGGCGACGCATTTGCGTTGTCTGTTTCGGCGCTCGCGTCCCACCTGTCTCTTCCTTTCTCTGCAGGCGGTGGGTACCGGGGCACAGCGACCTGAGAAGAGTGTGGGGCGGAAAACGCCTGGAAAAGGCATTCTGTGGAAATTACTTTTACTTCTTGGGAACGAGTGGCCAAGGGACACAGCTTTTTTTTTCAACCCGTCGTTCTCGTCAACACTTGGCGACACTCCCCCGACTCACGGGACTG

**Supplement 1.** The presentation of TGGT1_216200 construct sequences. The T7 promoter sequence is highlighted in yellow, the 5' UTR sequence is highlighted in blue, the TGGT1_216200 coding sequence is highlighted in red, and the 3' UTR sequence is displayed in gray font. We used the 300bp upstream and 300bp downstream sequences of the TGGT1_216200 gene for the 5' UTR and 3' UTR, respectively.
